# Unveiling the clinical and proteomic spectrum of relapsing polychondritis with respiratory involvement

**DOI:** 10.3389/fimmu.2026.1866652

**Published:** 2026-07-17

**Authors:** Xia Fang, Ximing Liao, Wei Gao, Wei Pei, Wujian Xu, Yuanyuan Wang, Yin Wu, Rongzhang Chen, Jiajun Ding, Muyun Wang, Xingyan Zhu, Heng Zhang, Dan-ni Ren, Qiang Li, Shaoyong Gao

**Affiliations:** 1 Department of Respiratory and Critical Care Medicine, Shanghai East Hospital, School of Medicine, Tongji University, Shanghai, China; 2 Department of Respiratory and Critical Care Medicine, Yingtan Hospital of Traditional Chinese Medicine, Yingtan, China; 3 Department of Respiratory and Critical Care Medicine, Yancheng Hospital of Traditional Chinese Medicine, Nanjing University of Chinese Medicine, Yancheng, China; 4 Academy of Integrative Medicine, College of Integrative Medicine, Fujian Key Laboratory of Integrative Medicine on Geriatric, Fujian University of Traditional Chinese Medicine, Fuzhou, China

**Keywords:** characteristics, complement system, neutrophil extracellular trap formation, proteomics, relapsing polychondritis, respiratory involvement

## Abstract

**Background:**

Relapsing polychondritis (RP) is a rare autoimmune disease characterized by recurrent inflammation and progressive damage to cartilage. Respiratory involvement, which has a relatively high misdiagnosis rate, is the leading cause of death in RP patients, and its underlying pathogenesis remains largely unknown.

**Methods:**

Seventy-seven RP patients with respiratory involvement were enrolled in this study. The clinical characteristics, including imaging features, bronchoscopic findings, and treatment, were systematically analyzed. Latent class analysis (LCA) was used to classify patients at first hospitalization according to their bronchoscopic findings, and the relevant clinical parameters were evaluated. A proteomic technique was applied to detect proteins in the peripheral serum of healthy controls (HCs) and 35 patients. The differentially expressed proteins between these two groups were analyzed and verified by Western blot.

**Results:**

Patients with initial respiratory involvement were less likely to have ear chondritis (14.29% vs 42.86%, *p* = 0.005), nasal chondritis (24.48% vs 46.43%, *p* = 0.048), and ocular involvement (16.33% vs 39.29%, *p* = 0.025). Patients can be classified into three subgroups by LCA based on bronchoscopic manifestations. Patients in groups one and two are defined by cartilage damage of the central airway, with or without peripheral airway involvement, and require more non-invasive ventilation. Patients in group three are defined by cartilage damage of the entire airway, with a high frequency of ICU admission (*p* = 0.003), tracheostomy (*p <*0.001), and rescue (*p* = 0.001). The expression of complement- and neutrophil extracellular trap formation (NETs)-related proteins may participate in the pathogenesis of respiratory-involved RP.

**Conclusions:**

Clinical manifestations of respiratory-involved RP are nonspecific, and recognition of imaging and bronchoscopic features can facilitate the diagnosis. Patient stratification based on the patterns of airway involvement can help guide airway management strategies. Additionally, abnormalities of the complement system and NETs may play a role in its pathogenesis.

## 1 Introduction

Relapsing polychondritis (RP) is a rare immune-mediated systemic disease that has a predilection for causing damage to cartilaginous tissue or tissue rich in proteoglycans (1). Respiratory involvement in RP, which primarily affects the larynx, trachea, and bronchi, is not only a common complication but also a major cause of mortality (2, 3). Patients with respiratory involvement often present with symptoms such as cough, wheezing, and dyspnea. These symptoms progressively worsen as immune-mediated inflammation causes cartilage destruction, leading to airway malacia, collapse, or even obstruction (4, 5). The diagnosis of RP with initial respiratory involvement, especially at an early stage, remains a major clinical challenge. Because the clinical manifestations are highly similar to those of common chronic respiratory diseases such as asthma and chronic obstructive pulmonary disease (COPD), this frequently results in misdiagnosis or delayed diagnosis (6). Therefore, analyzing the clinical feature spectrum of RP patients with respiratory involvement is of great clinical significance.

To date, the pathogenesis of RP remains largely unknown. A small body of evidence suggests that exposure to autoantigens, such as type II collagen and matrilin-1, which are specific to cartilaginous tissues (7), and to misfolded proteins may play pivotal roles in initiating the autoimmune response, leading to inflammation and subsequent cartilage damage (8). Notably, respiratory involvement in RP, which occurs in approximately half of patients and can lead to life-threatening complications (9), has a poorly understood underlying pathogenesis. Proteomics has emerged as a powerful tool in unraveling the complex molecular mechanisms of autoimmune and inflammatory diseases (10). It enables high-throughput detection and analysis of global protein expression profiles, facilitating the identification of novel biomarkers and pathogenic molecules.

This study retrospectively analyzes the clinical features of RP patients with respiratory involvement, aiming to improve their identification. Additionally, this study aims to explore the protein expression profiles of patients with respiratory-involved RP using proteomic approaches, thus elucidating the underlying pathogenesis of this subset of patients.

## 2 Methods

### 2.1 Patients

A total of 77 RP patients with respiratory involvement were recruited from our institution between 2020 and 2025. Inclusion criteria were as follows (1): Patients were older than 18 years of age. (2) RP diagnosis was based on the criteria of McAdam, Damiani and Levine, or Michet. (3) Respiratory involvement was confirmed by chest CT and bronchoscopy. Exclusion criteria were as follows: (1) Patients had other autoimmune diseases, tumors, hematological disorders, or immunodeficiency diseases. (2) Patients did not have complete follow-up data. Follow-up, either via hospital readmission or telephone contact, was conducted every three months after initial discharge, including symptom assessment, detection of inflammatory markers, evaluation of medication regimens, and monitoring of adverse drug reactions. CT scans of the larynx and chest, pulmonary function tests, and bronchoscopy were repeated every three to six months. In this study, four patients were excluded due to missing some of the aforementioned follow-up data. Among them, two patients in the airway-onset group were lost to follow-up at seven and eleven months after initial treatment at our center, respectively, and one patient failed to complete the follow-up at nine months. One patient in the non-airway-onset group failed to complete follow-up at 12 months. The flowchart is shown in **Supplementary Figure 1**.

For the proteomic analysis, 10 healthy controls (HCs) and 35 patients were recruited. This study was carried out in accordance with the Declaration of Helsinki of the World Medical Association, was approved by the Ethics Committee of Shanghai East Hospital (2026YS-007), and written informed consent was obtained from all patients.

### 2.2 LCA analysis

Latent class analysis (LCA) was performed to identify clinical subgroups of patients with respiratory-involved RP based on predefined bronchoscopic variables (11). LCA is a model-based, unbiased classification method that classifies individuals into mutually exclusive latent subgroups according to shared clinical patterns reflected by a set of categorical variables (13). The variables included in the LCA model were bronchoscopic manifestations, including vocal cord edema, laryngeal cartilage deformation, subglottic stenosis, mucosal hypertrophy, disappearance of cartilaginous rings (in the trachea and bronchi above and below the lobar level), and airway stenosis caused by malacia (in the trachea and bronchi above and below the lobar level). All input variables were coded as binary variables according to the presence or absence of each bronchoscopic manifestation. Model fit was evaluated using Akaike’s information criterion (AIC). Models with two to five latent classes were fitted, and the model with the lowest AIC was selected as the final model. The resulting subgroups were then labeled according to the predominant anatomical pattern of airway involvement in each class.

### 2.3 Proteomics test

#### 2.3.1 Sample preparation

Peripheral blood was collected from 10 HCs and 35 patients before initial treatment and centrifuged at 1,300×*g* at 4 °C for 10 min. The serum was then transferred to a new 1.5 mL EP tube for subsequent experiments. First, cellular debris in the serum sample was removed by centrifugation at 12,000×*g* at 4 °C for 10 min, and the supernatant was transferred to a new centrifuge tube. Then, the top 14 high-abundance proteins were removed using the Pierce™ Top 14 Abundant Protein Depletion Spin Columns Kit (Thermo Fisher Scientific). The protein concentration was determined using a BCA kit according to the manufacturer’s instructions. For digestion, the protein solution was reduced with 5 mM dithiothreitol for 30 min at 56 °C and alkylated with 11 mM iodoacetamide for 15 min at room temperature in the dark. The protein sample was then diluted by adding 100 mM TEAB until the urea concentration was less than 2 M. Finally, trypsin was added at a 1:50 trypsin-to-protein mass ratio for the first overnight digestion and at a 1:100 trypsin-to-protein mass ratio for a second 4-hour digestion. The peptides were then desalted using a C18 SPE column.

#### 2.3.2 LC–MS/MS analysis

The tryptic peptides were dissolved in solvent A and directly loaded onto a homemade reversed-phase analytical column (25 cm long, 100 μm i.d.). The mobile phase consisted of solvent A (0.1% formic acid, 2% acetonitrile in water) and solvent B (0.1% formic acid, 90% acetonitrile in water). Peptides were separated using the following gradient: 0 min–16 min, 6%–20%B; 16 min–24 min, 20%–32% B; 24 min–27 min, 32%–80% B; 27 min–30 min, 80% B, all at a constant flow rate of 500 nL/min on an EASY-nLC 1200 UPLC system (Thermo Fisher Scientific). The separated peptides were analyzed using an Orbitrap Exploris 480 equipped with a nano-electrospray ion source. The electrospray voltage applied was set to 2,100 V. Precursors and fragments were analyzed using the Orbitrap detector. The full MS scan resolution was set to 30,000 for a scan range of 350 m/z–1,050 m/z. The MS/MS scan used a first mass of 200.0 m/z with a resolution of 45,000. The HCD fragmentation was carried out at normalized collision energies (NCE) of 25%, 30%, and 35%. The automatic gain control (AGC) target was set to 3 × 10^6^, with a maximum injection time of auto.

#### 2.3.3 Bioinformatics analysis

Functional enrichment was performed using Fisher’s exact test, and functional terms with fold enrichment >1.5 and a *p*-value <0.05 were considered significant. Enrichment-based clustering was performed based on the functional enrichment of differentially expressed proteins. The clustering relationships were visualized using a heatmap generated by the heatmap function in the R package ComplexHeatmap (12). Protein–protein interaction networks were searched against the STRING database, and a confidence score >0.7 (high confidence) was considered to indicate a significant interaction (13). The interaction network from STRING was visualized using the R package visNetwork.

### 2.3 Western blot analysis

Plasma samples were diluted 1:10 in a loading buffer containing 5% β-mercaptoethanol. The samples were then heated in a boiling water bath for 5 min prior to SDS-PAGE analysis. Samples were separated on 4%–12% Bis-Tris gels via electrophoresis at 110 V for 90 min. Subsequently, the separated proteins were electrotransferred onto polyvinylidene fluoride (PVDF) membranes at 330 mA for 90 min. The membranes were blocked with 5% non-fat dry milk at room temperature for 1 h. The membranes were then incubated with primary antibodies (anti-C3: 1:2,000, EPR19394, Abcam; anti-C5: 1:1,000, EPR23940-3, Abcam; anti-MPO: 1:1,000, EPR20257, Abcam) overnight at 4 °C. After three washes with TBST, the membranes were incubated with the corresponding secondary antibodies for 1 h at room temperature. Finally, an ECL chemiluminescent substrate was applied, and target protein bands were detected and visualized using autoradiographic film.

### 2.4 Statistical analysis

Continuous variables were expressed as medians (25th–75th percentile range) or means ± standard deviations (mean ± SD). Comparisons between groups were performed using the Wilcoxon test, Student’s t-test, or Welch’s t-test. The chi-square test was used for categorical variables, and categorical variables were presented as frequencies and percentages. For the analysis of differentially expressed proteins across groups, raw proteomic relative expression values were presented as means ± SEM. To reduce the influence of skewed data distributions, the original relative expression values were log_2_-transformed before statistical analysis. Overall differences among three or more groups were assessed using one-way ANOVA. If the overall difference was statistically significant, *post hoc* multiple comparisons were further performed using Tukey’s test among groups or Dunnett’s test for comparisons with healthy controls (HCs). All tests were two-sided, and *p*-values <0.05 were considered statistically significant. The data were analyzed using GraphPad Prism (10.4.2) and IBM SPSS Statistics (27.0.1).

## 3 Results

### 3.1 The clinical features of the study cohort

Among the 77 patients, 35 (45.5%) were male and 42 (54.5%) were female, with a median age at diagnosis of 54 years (**Table 1**). Patients were divided into two subgroups based on whether airway involvement was the initial symptom. Compared with the non-airway-onset group, patients with airway onset were less likely to have ear and nasal chondritis, as well as ocular involvement (**Table 1**). Notably, the median time to diagnosis in RP patients with airway onset was 180 days, which was longer than that in the non-airway-onset group (150 days), although the difference was not statistically significant. Among these patients, two developed respiratory chondritis after receiving immune checkpoint inhibitor treatment, with rare involvement of other organs.

**Table 1:** Clinical characteristics of RP patients with respiratory involvement.

Characteristic	Airway onset (n = 49)	Non-airway onset (n = 28)	Total (n = 77)	P-value
Gender				0.076
–Male, no. (%)	26 (53.06)	9 (32.14)	35 (45.45)	
–Female, no. (%)	23 (46.94)	19 (67.86)	42 (54.55)	
Age at diagnosis				0.729
–<18 years, no. (%)	1 (2.04)	0 (0.00)	1 (1.30)	
–18–65 years, no. (%)	36 (73.47)	23 (82.14)	59 (76.62)	
>65 years, no. (%)	12 (24.49)	5 (17.86)	17 (22.08)	
Ear chondritis, no. (%)	7 (14.29)	12 (42.86)	19 (24.68)	0.005
Nasal chondritis, no. (%)	12 (24.49)	13 (46.43)	25 (32.47)	0.048
Laryngeal chondritis, no. (%)	12 (24.49)	14 (50.00)	26 (33.77)	0.023
Respiratory chondritis, no. (%)	49 (100.00)	28 (100.00)	77 (100.00)	/
Ocular involvement, no. (%)	8 (16.33)	11 (39.29)	19 (24.68)	0.025
Joint involvement, no. (%)	10 (20.41)	12 (42.86)	22 (28.57)	0.036
Cardiovascular involvement, no. (%)	1 (2.04)	1 (3.57)	2 (2.60)	1.000
ICIs-induced RP, no. (%)	2 (4.08)	0 (0.00)	2 (2.60)	0.531
Diagnostic delay, median (IQR) years	180.00 (52.50–409.50)	150.00 (30.00–313.25)		0.328

Data are presented as n, mean ± SD or n (%); ICIs, immune checkpoint inhibitors.

### 3.2 The CT scan and bronchoscopic findings

All patients underwent a dynamic chest CT scan. The imaging features investigated included subglottic stenosis, thickening of the tracheal wall, tracheal/bronchial calcification, tracheal/bronchial stenosis caused by malacia, bronchiectasis, and gas trapping (**Supplementary Figure 2**). Of these, thickening of the tracheal wall and tracheal/bronchial stenosis caused by malacia were the most common findings (**Supplementary Table 1**). In addition, all patients at our center underwent bronchoscopic examination (**Supplementary Figure 3**). Under endoscopic observation, the majority of patients presented with mucosal hypertrophy (89.6%), disappearance of the bronchial cartilaginous rings (97.4%), and bronchial stenosis caused by malacia in the bronchi above the lobar level (97.4%) (**Supplementary Table 2**).

### 3.3 Comparison of different subgroups of RP patients with respiratory involvement

**Figure 1:**
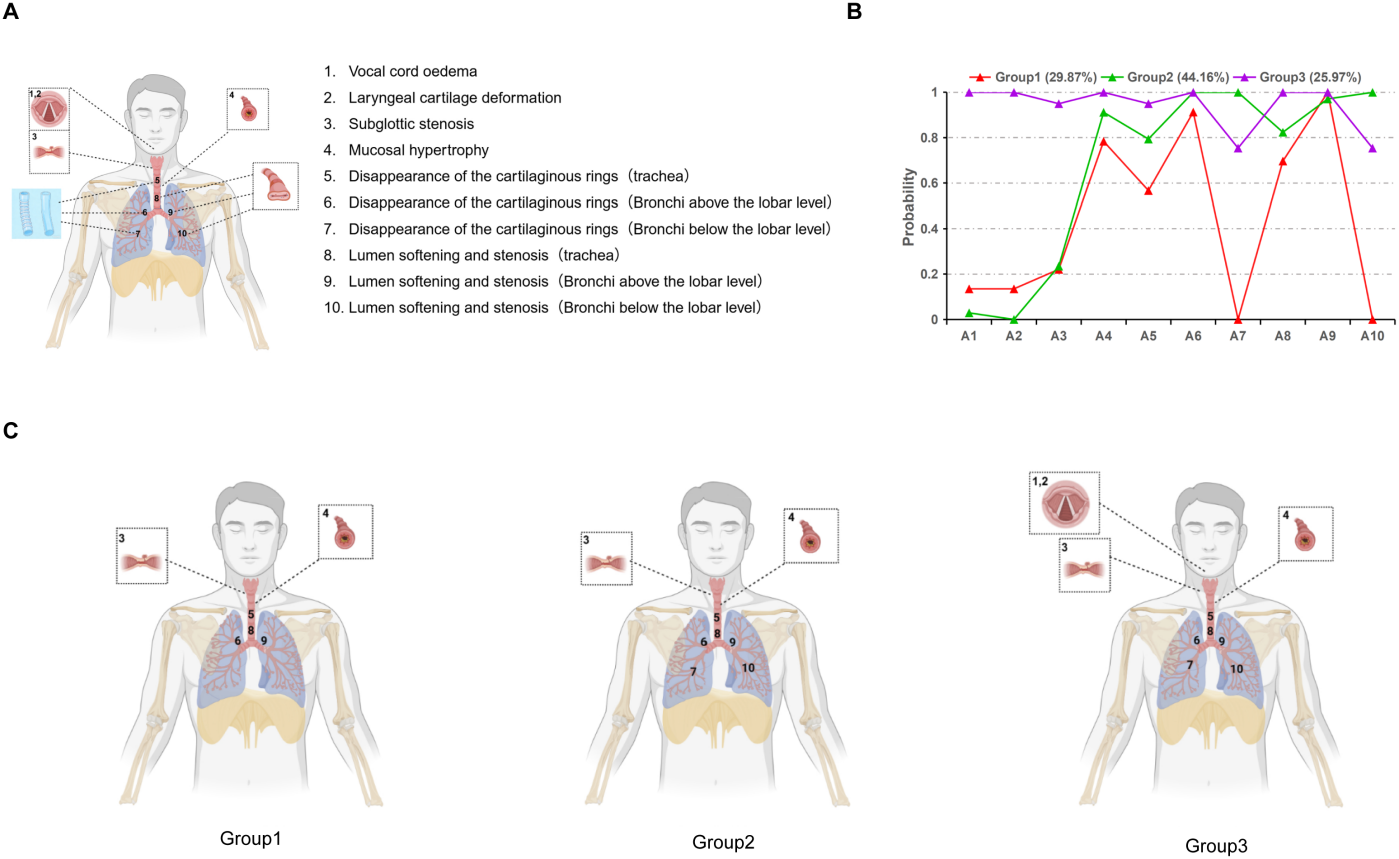
The subgroups of RP patients with respiratory involvement based on bronchoscopic manifestations. **(A)** Variables used in latent class analysis modeling. **(B, C)** Illustrations of the three subgroups of RP patients with respiratory involvement identified by latent class analysis. This figure was generated using BioRender.

Latent class analysis classified three subgroups of RP patients with respiratory involvement at first hospitalization based on bronchoscopic manifestations (**Figures 1A, B**). The first group was defined by cartilage damage of the trachea and main bronchi (n = 23 [29.87%], **Figure 1C**). The second group was characterized by cartilage damage of the trachea, proximal (above lobar) and distal (below lobar) bronchi (n = 34 [44.16%], **Figure 1C**). The third group was defined by cartilage damage of the larynx, trachea, proximal and distal bronchi (n = 20 [25.97%], **Figure 1C**). Compared with the other two groups, patients in the third group exhibited higher rates of ICU admission, tracheostomy, and rescue therapy, suggesting that RP patients with entire airway involvement require intensive care and enhanced respiratory support. In addition, patients in the first and second groups were more likely to require non-invasive ventilation (**Table 2**). Furthermore, the results showed no significant difference in survival time among these subgroups. There were two deaths in group one, three deaths in group two, and one death in group three. Except for one patient in group one who died by suicide following depression, all other patients died due to the disease itself (**Supplementary Figure 4**; **Table 2**).

**Table 2:** Clinical characteristics of three subgroups of RP patients with respiratory involvement.

Parameter	All patients (n = 77)	Group 1 (n = 23)	Group 2 (n = 34)	Group 3 (n = 20)	P-value
Variables included in latent class analysis					
–Vocal cord edema, no. (%)	24 (31.17)	3 (13.04)_a_	1 (2.94)_a_	20 (100.00)_b_	<0.001
–Laryngeal cartilage deformation, no. (%)	23 (29.87)	3 (13.04)_a_	0 (0.00)_a_	20 (100.00)_b_	<0.001
–Subglottic stenosis, no. (%)	32 (41.56)	5 (21.74)_a_	8 (23.53)_a_	19 (95.00)_b_	<0.001
–mucosal hypertrophy, no. (%)	69 (89.61)	18 (78.26)_a_	31 (91.18)_a_	20 (100.00)_a_	0.058
–Disappearance of the cartilaginous rings					
–trachea, no. (%)	59 (76.62)	13 (56.52)_a_	27 (79.41)_a,b_	19 (95.00)_b_	0.011
–Bronchi above the lobar level, no. (%)	75 (97.40)	21 (91.30)_a_	34 (100.00)_a_	20 (100.00)_a_	0.151
–Bronchi below the lobar level, no. (%)	49 (63.64)	0 (0.00)_a_	34 (100.00)_b_	15 (75.00)_c_	<0.001
–Lumen softening and stenosis					
–trachea, no. (%)	64 _(_83.12_)_	16 (69.57)_a_	28 (82.35)_a,b_	20 (100.00)_b_	0.020
–Bronchi above the lobar level, no. (%)	76 (98.70)	23 (100.00)_a_	33 (97.06)_a_	20 (100.00)_a_	1.000
–Bronchi below the lobar level, no. (%)	49 (63.64)	0 (0.00)_a_	34 (100.00)_b_	15 (75.00)_c_	<0.001
Demographic characteristics					
–Male, no. (%)	35 (45.45)	12 (52.17)_a_	20 (58.82)_a_	3 (15.00)_b_	0.006
–Female, no. (%)	42 (54.55)	11 (47.83)_a_	14 (41.18)_a_	17 (85.00)_b_	0.006
–Age at diagnosis, median (IQR) years	57.00 (47.00–64.0)	58.00 (48.00–71.00)	59.50 (51.75–65.50)	52.50 (42.00–59.25)	0.060
–Diagnostic delay, median (IQR) years	180.00 (30.00–362.50)	60.00 (30.00–330.00)	210.00 (30.00–445.50)	192.50 (90.00–259.50)	0.505
Others					
–ICU admission, no. (%)	26 (33.77)	5 (21.74)_a_	8 (23.53)_a_	13 (65.00)_b_	0.003
–Endotracheal intubation, no. (%)	6 (7.79)	2 (8.70)_a_	1 (2.94)_a_	3 (15.00)_a_	0.270
–Tracheostomy, no. (%)	17 (22.08)	4 (17.39)_a_	1 (2.94)_a_	12 (60.00)_b_	<0.001
–Rescue, no. (%)	22 (28.57)	4 (17.39)_a_	6 (17.65)_a_	12 (60.00)_b_	0.001
–NIPPV, no. (%)	21 (27.27)	3 (13.04)_a_	12 (35.29)_a_	6 (30.00)_a_	0.172
–Alive, no. (%)	71 (92.21)	21 (91.30)_a_	31 (91.18)_a_	19 (95.00)_a_	1.000
–Dead, no. (%)	6 (7.79)	2 (8.70)_a_	3 (8.82)_a_	1 (5.00)_a_	1.000

(Rank sum test) Each subscript letter a, b indicates a subset of teaching method categories, and at the 0.05 level, there is no significant difference in the column proportions among these categories.

### 3.4 Conventional treatment and biotherapy

Most patients were treated with a combination of glucocorticoids (methylprednisolone or prednisone) and disease-modifying antirheumatic drugs (DMARDs) (**Supplementary Table 3**). Thirty-two patients received biologic therapy, with the majority receiving tocilizumab. Four of these patients received two biologic agents sequentially (**Supplementary Table 4**). Of these, most patients achieved relief from extrapulmonary symptoms. However, only nine patients remained in remission, with respiratory chondritis controlled by tofacitinib or tocilizumab (**Supplementary Table 4**). In addition, our cohort showed that the incidence of pneumonia was higher in patients treated with adalimumab and baricitinib.

### 3.5 Proteomic analysis of RP patients with respiratory involvement

**Figure 2:**
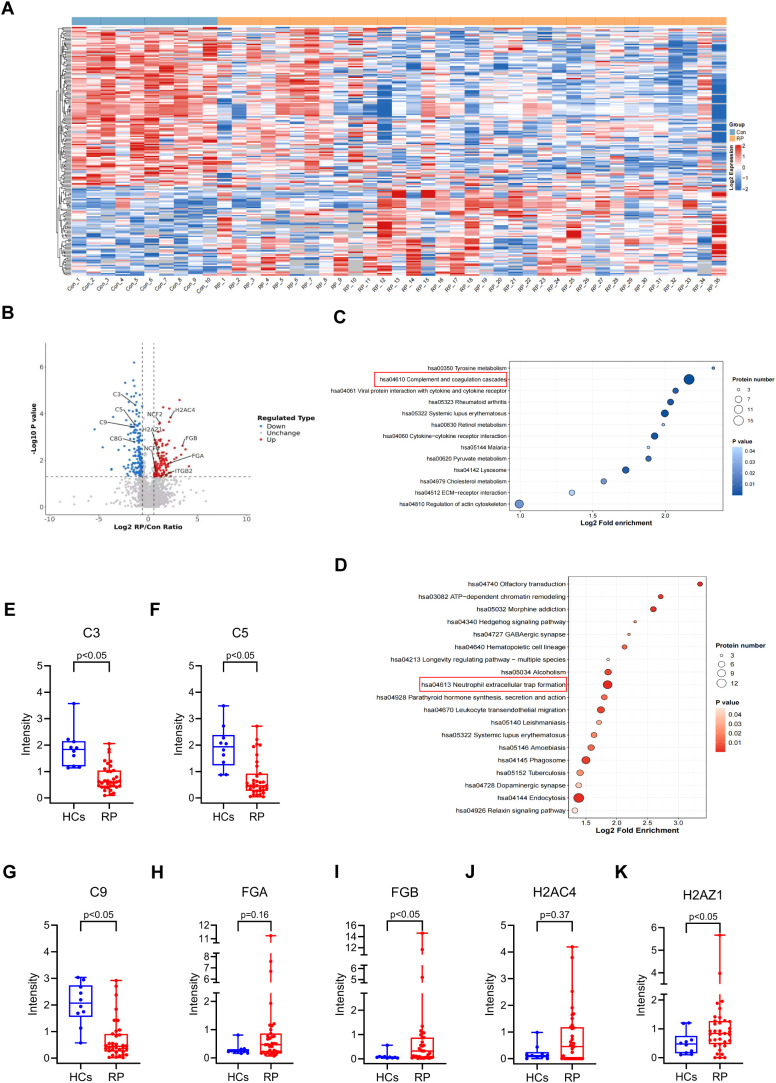
Protein expression profile of RP patients with respiratory involvement. **(A, B)** Heatmap **(A)** and volcano plots **(B)** of significantly differentially expressed proteins in serum from healthy controls (HCs) (n = 10) and RP patients with respiratory involvement (n = 35). **(C, D)** Downregulated **(C)** and upregulated **(D)** proteins contributing to the most representative enriched Kyoto Encyclopedia of Genes and Genomes (KEGG) pathway terms are highlighted by the red column. **(E–G)** The expression of C3, C5, and C9 in HCs and patients. **(H–K)** The expression of FGA, FGB, H2AC4, and H2AZ1 in HCs and patients.

To explore the underlying pathogenesis of respiratory-involved RP, high-throughput proteomic analysis was performed on 35 patients and 10 healthy controls (HCs). As shown in **Figure 2A**, the expression pattern exhibited a distinct difference between patients and HCs. PCA further confirmed a relatively clear separation between these two groups (**Supplementary Figure 5**). A total of 286 proteins were differentially expressed in patients compared with HCs, including 118 that were upregulated and 168 that were downregulated (**Figure 2B**). Notably, Kyoto Encyclopedia of Genes and Genomes (KEGG) pathway enrichment analysis revealed that proteins with significantly downregulated expression were primarily involved in complement and coagulation cascades (**Figure 2C**), including C3, C5, and C9, etc. (**Figures 2E–G**). Meanwhile, the upregulated proteins were predominantly involved in neutrophil extracellular trap formation (NETs) (**Figures 2D, H–K**). Furthermore, we explored the relationships between complement system abnormalities, NETs, and the severity of airway damage in RP patients. The proteomic results showed that the expression levels of C3, C5, and C9 showed no significant statistical differences among group 1 (central airway involvement), group 2 (combined central and peripheral airway involvement), and group 3 (entire-airway involvement) (**Supplementary Figures 6A–C**), nor did the expression levels of NETs-related proteins (**Supplementary Figures 6D–F**). These results suggest that alterations in the complement system and NETs may be associated with the pathogenesis of RP with respiratory involvement, while their correlation with the severity of airway injury remains to be further investigated.

Subsequently, we analyzed differentially expressed proteins (DEPs) between patients with airway onset and those with non-airway onset (**Supplementary Figure 7A**). A total of 92 DEPs were identified, consisting of 75 upregulated and 17 downregulated proteins. Functional enrichment analysis of these DEPs revealed that upregulated proteins in the airway onset group were predominantly enriched in the calcium and the IL-17 signaling pathways (**Supplementary Figure 7B**), including PDGFB, VEGFD, MMP1, and CXCL5, relative to the non-airway onset group (**Supplementary Figures 7C–F**). Likewise, DEP profiling across the three subgroups was also analyzed (**Supplementary Figure 8A**). A total of 147 DEPs were identified, consisting of 81 upregulated and 66 downregulated proteins. Specifically, the upregulated proteins between group one and group two were mainly enriched in the endocytosis pathway (**Supplementary Figure 8B**), with group one showing higher expression levels of SNX2 and SNX5 compared with group two (**Supplementary Figures 8C, D**). The upregulated DEPs between group two and group three were mainly enriched in the Ras and MAPK pathways (**Supplementary Figure 8B**). Most downregulated DEPs were identified in the comparison between group two and group three. These downregulated DEPs were enriched in the endocytosis pathway (**Supplementary Figure 8B**), with group two showing lower expression levels of SNX6 and ACAP1 than group three (**Supplementary Figures 8E, F**). In summary, these findings suggest that the aforementioned DEPs may be associated with the pathogenesis of airway damage in RP patients. However, due to the limited sample size in each group, further validation is needed in a larger patient cohort.

### 3.6 Verify screened proteins and explore their clinical relevance

**Figure 3:**
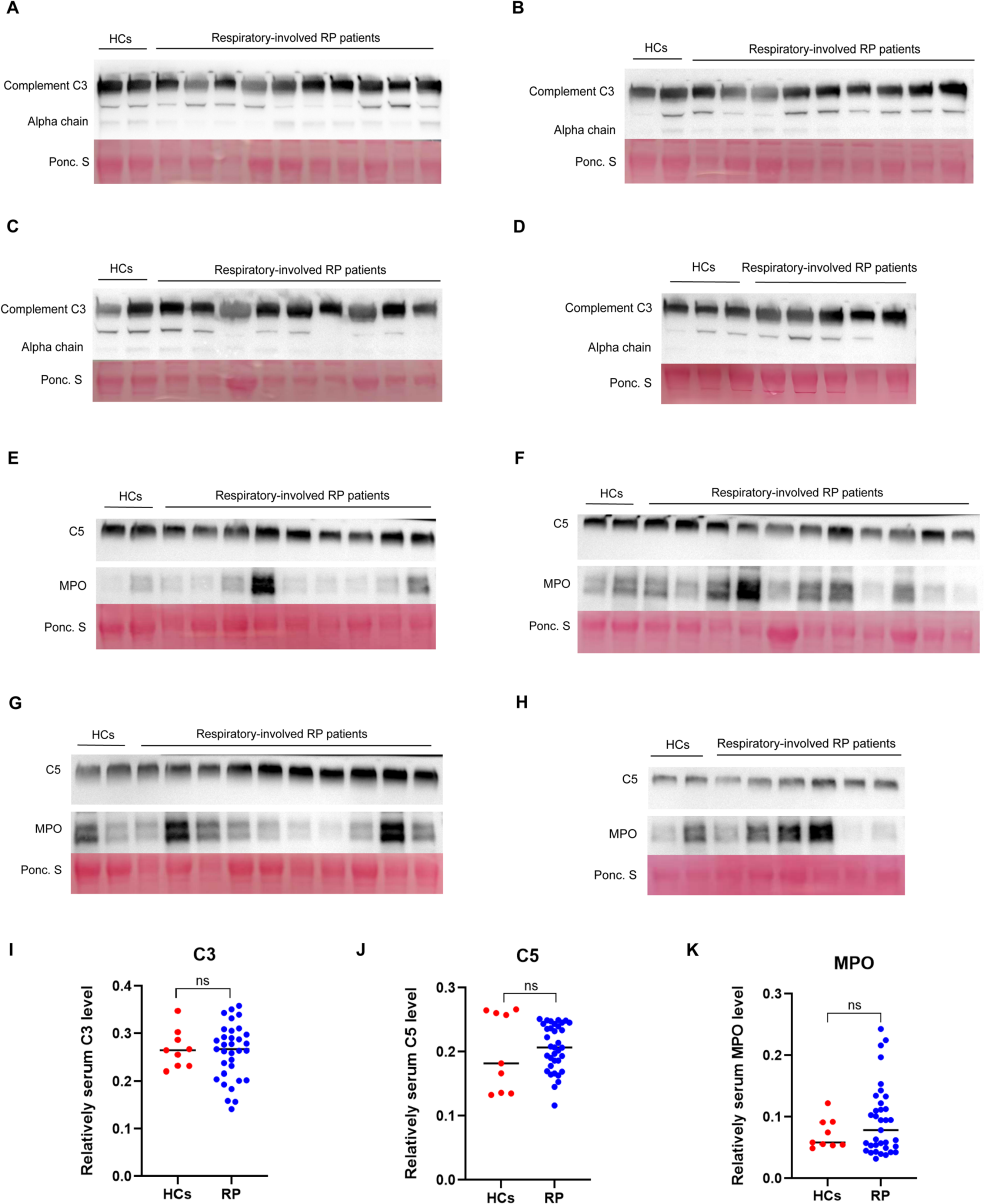
Expression of C3, C5, and MPO in patients with respiratory involvement. C3 **(A–D)** and C5 **(E–H)** exhibited reduced expression in some patients, whereas MPO **(E–H)** expression was elevated in certain patients. **(I–K)** Quantitative analysis of C3 **(I)**, C5 **(J)**, and MPO **(K)** protein expression.

Next, due to the significant differences in the expression of complement system components and NETs-related proteins in RP patients with airway involvement, we detected the expression levels of C3, C5, and myeloperoxidase (MPO, a canonical biomarker of NET formation (14)) in the peripheral serum of the enrolled patients. As shown in **Figure 3**, although decreased expression of C3 and C5 was not observed in all patients, a downward trend of these proteins was identified in some patients. Similarly, MPO expression was markedly upregulated in several patients. Collectively, these findings suggest that the complement system and NETs may play a partial role in the pathogenesis of respiratory-involved RP.

To identify which clinical characteristics were associated with distinct expression levels of C3, C5, and MPO proteins among the patients, we subsequently performed stratified analyses of the expression levels of these proteins. The results showed that there were no significant statistical differences in clinical characteristics between patients with distinct expression of C3, C5 and MPO and those with unchanged expression, including sex, age at diagnosis, the presence of systemic symptoms (e.g., ear, nasal and laryngeal chondritis, ocular and joint involvement), the degree of airway involvement and inflammatory markers (**Supplementary Table 5**). The above findings may be attributable to limited sample size and inter-individual heterogeneity. Further studies including larger cohorts of RP patients with respiratory involvement are needed to validate the expression of these proteins and its relationship with clinical profiles and disease severity.

## 4 Discussion

As one of the most severe complications of RP, respiratory chondritis is the leading cause of death in these patients, and it is frequently misdiagnosed or underdiagnosed due to the absence of specific symptoms. In addition, the underlying pathogenesis of respiratory-involved RP remains largely unknown. Our study systematically analyzed the clinical characteristics of RP patients with respiratory involvement and explored the potential pathogenesis of this population. The present study showed that RP patients who primarily presented with airway involvement at disease onset tended to have relatively fewer systemic manifestations. Their imaging findings were dominated by the thickening of the tracheal wall and tracheal/bronchial stenosis caused by malacia. Among these patients, those with central airway involvement (with or without peripheral airway involvement) required non-invasive ventilation treatment, whereas patients with entire airway involvement need more critical care and management. These findings not only highlight the importance of imaging features and bronchoscopic examinations in the diagnosis of RP patients with respiratory involvement but also indicate that different patterns of airway involvement may require different airway management strategies. In addition, our results indicated that the dysregulation of the complement system and NETs may play a role in the pathogenesis of respiratory-involved RP.

Previously, a study by Zhai et al. demonstrated that RP patients presenting who initially presented with respiratory involvement had less frequent systemic involvement (e.g., ocular and auricular involvement) and were mainly complicated by costochondritis (3). In our study, we also found that patients with initial respiratory involvement had relatively fewer extrapulmonary systemic manifestations. Moreover, due to the lack of specific respiratory symptoms and systemic signs, these patients generally experienced a longer time to a definitive diagnosis (15, 16). Therefore, analyzing the clinical features of RP patients with respiratory involvement, including symptoms, CT imaging, and bronchoscopic findings, may facilitate an in-depth understanding of this disease and assist clinicians, particularly pulmonologists, in making a prompt diagnosis and initiating appropriate treatment. Previous studies demonstrated that the CT imaging characteristics of respiratory-involved RP included thickening of the tracheal wall, tracheal/bronchial collapse, and stenosis (17). Similarly, our study showed that the CT manifestations in these patients were more frequently characterized by tracheal/bronchial stenosis caused by malacia and thickening of the tracheal wall. In fact, it is often difficult to determine the presence of respiratory chondritis in patients with respiratory predominance simply by combining clinical symptoms with CT imaging findings. In such cases, further diagnostic evaluations are necessary. Accumulating evidence suggests that PET-CT plays a crucial role in the early diagnosis of patients with respiratory-involved RP (18). Our data indicated that 18.2% of patients were ultimately diagnosed with RP after PET-CT examinations, suggesting that PET-CT may serve as a valuable adjunct in early diagnosis.

Notably, bronchoscopic examination plays an irreplaceable role in the early diagnosis of RP patients with respiratory involvement, as it allows for the direct visualization and assessment of airway damage (19). Previous studies indicated that bronchoscopic test may be associated with potential risks, such as worsening laryngeal edema, airway spasm, and the need for tracheal intubation (20). In our study, five patients with severe airway involvement developed hypoxia during bronchoscopy and required emergency endotracheal intubation. Based on our single-center experience, bronchoscopy may be considered when a patient presents with an unexplained cough or wheezing, is suspected of having RP, and lacks other systemic manifestations. Under direct visualization, bronchoscopy can be used to observe and assess mucosal edema, collapse of the cartilaginous rings, and dynamic tracheobronchomalacia. Additionally, bronchoscopy can serve as an assessment tool for disease monitoring and for dynamically evaluating the extent of airway cartilage damage, as well as the degree of chondromalacia and collapse. For patients whose dyspnea is exacerbated by retained secretions, bronchoscopic local therapy may also be performed to relieve respiratory symptoms. However, it is important to note that in patients with severe laryngeal destruction or severe airway collapse, bronchoscopy carries risks of inducing laryngeal edema and obstruction, as well as intraoperative hypoxia requiring emergency intubation. Therefore, bronchoscopy in RP patients with airway involvement must be preceded by a thorough preoperative evaluation, incorporating the patient’s symptoms, blood gas analysis, pulmonary function tests, and imaging findings from laryngopharyngeal and chest CT findings. When necessary, laryngoscopy should be performed first to assess the condition of the larynx. Following a comprehensive evaluation, the procedure should be carried out with sedation administered by an experienced anesthesiologist and bronchoscopy performed by an experienced endoscopist, with close intraoperative monitoring of the patient’s heart rate, respiration, and oxygen saturation. Previously, Ferrada et al. classified the clinical characteristics of RP patients using latent class analysis (LCA) to optimize the early diagnosis of RP (6). However, no classification system has been established for patients with respiratory involvement. In the present study, we used LCA to subgroup these patients during their first hospitalization based on bronchoscopic findings and found that RP patients with entire airway involvement had a higher rate of ICU admission and more frequent rescue interventions. Our study suggests that RP patients with respiratory involvement who present with marked dyspnea and hoarseness, severe destruction of the laryngeal cartilage observed endoscopically, and concurrent central and peripheral airway involvement (whole-airway involvement) should receive intensive care and advanced respiratory support. Tracheostomy should be considered if necessary. For patients with central airway involvement, with or without peripheral airway involvement, non-invasive respiratory support should be provided in addition to local airway interventions. Of course, systemic therapy is indispensable and serves as the primary measure for disease control, requiring multidisciplinary clinical decision-making by rheumatologists.

Currently, the management of respiratory-involved RP remains a significant clinical challenge, as there is a lack of effective regimens capable of halting or reversing the destruction of airway cartilage (21). Previous studies indicated that biologic agents may have favorable efficacy in the treatment of RP patients with respiratory involvement (22, 23). Our study investigates that, compared with its effect on controlling respiratory chondritis, biologic therapy has a more favorable effect on extrapulmonary inflammatory symptoms. Moreover, even when some patients received biologic therapy before the occurrence of respiratory symptoms, airway damage still occurred at a later stage. Therefore, it is necessary to explore the pathogenesis of respiratory-involved RP. In the present study, downregulation of complement C3 and C5 was found in some RP patients with respiratory involvement, indicating that the complement system may play a role in the pathogenesis of respiratory-involved RP. Similarly, a previous study showed that complement component C4B was significantly reduced in the peripheral blood of RP patients but markedly deposited in airway cartilage (24), suggesting complement activation and excessive consumption in RP patients. Based on these findings, we hypothesize that excessive complement activation mediates and exacerbates local cartilage inflammation. Furthermore, persistent complement consumption impairs the system’s ability to eliminate immune complexes and apoptotic cells, triggering recurrent complement activation to establish a vicious cycle, ultimately leading to recurrent cartilage inflammation and structural damage to the cartilage matrix and cells. Further prospective investigations are warranted to validate this mechanistic hypothesis. Additionally, previous studies found substantial neutrophil infiltration in the soft tissues surrounding nasal and auricular cartilage in RP patients (25, 26). In line with our proteomic results, which also revealed upregulation of NETs-related proteins in the peripheral blood of RP patients, these findings suggest that NETs may also be involved in driving cartilage inflammation and autoimmunity.

The limitation of our study is that it is a single-center study. Although RP is a rare disease, multicenter studies with larger sample sizes are still needed to further validate the role of the complement system and NETs in the pathogenesis of RP with respiratory involvement. In addition, there is currently a lack of ideal animal models for studying respiratory-involved RP. In the future, it is necessary to establish animal models that closely mimic the clinical presentation to better elucidate the underlying mechanisms of this disease and evaluate potential therapeutic agents.

## Data Availability

The raw date of proteomics presented in the study are publicly available. This data can be found here: https://proteomecentral.proteomexchange.org/cgi/GetDataset?ID=PXD080705, the access number: PXD080705.

## Supplementary material

Supplementary Table 1 The features of CT scan findings.

Supplementary Table 2 The bronchoscopic findings.

Supplementary Table 3 Therapy.

Supplementary Table 4 Control rate of biological agents.

Supplementary Table 5 Clinical relevance of C3, C5 and MPO.

Supplementary Figure 1 Flow chart of patient enrollment in this study.

Supplementary Figure 2 The manifestation of CT scan findings. **(A)** Subglottic stenosis. **(B)** Thickening of the tracheal wall. **(C)** Bronchial calcification. **(D, E)** Tracheal **(D)** and bronchial **(E)** stenosis caused by malacia. **(F)** Local gas trapping in the right lower lung (white arrow).

Supplementary Figure 3 The bronchoscopic findings. **(A)** Laryngeal edema and stenosis. **(B)** Subglottic stenosis. **(C)** Mucosal hypertrophy. **(D–F)** Disappearance of the cartilage rings in the trachea and bronchi, and luminal stenosis caused by tracheobronchial malacia.

Supplementary Figure 4 Kaplan-Meier curves for subgroups. The survival analysis of patients in the three groups.

Supplementary Figure 5 PCA illustration. The PCA illustration between HCs (con) and RP patients with respiratory involvement.

Supplementary Figure 6 The expression of candidate proteins in subgroups of RP patients with respiratory involvement. **(A–C)** The expression of C3, C5 and C9 in HCs and subgroups of patients. **(D–F)** The expression of FGA, FGB and H2AC4 in HCs and subgroups of patients.

Supplementary Figure 7 Protein expression profile of RP patients with airway onset and non-airway onset. **(A)** Heatmap of significantly differentially expressed proteins in the blood serum from HCs (n=10) and RP patients with airway onset (AO, n=21) and non-airway onset (NAO, n=14). **(B)** KEGG pathway enrichment analysis of differentially expressed proteins among these groups. Pathways enriched by upregulated proteins in airway onset relative to non-airway onset are marked in red. **(C-F)** The expression of PDGFB, VEGFD, MMP1 and CXCL5 in patients with airway onset (AO) and non-airway onset (NAO).

Supplementary Figure 8 Protein expression profiles of patient subgroups. **(A)** Heatmap of significantly differentially expressed proteins in the blood serum from HCs and patient subgroups. **(B)** KEGG pathway enrichment analysis of differentially expressed proteins among these groups. Pathways enriched by upregulated and downregulated proteins among these subgroups are marked in red. **(C, D)** The expression of SNX2 and SNX5 in group one and group two. **(E, F)** The expression of SNX6 and ACAP1 in group two and group three.
