# Correction: Unveiling the clinical and proteomic spectrum of relapsing polychondritis with respiratory involvement

**DOI:** 10.3389/fimmu.2026.1943834

**Published:** 2026-07-30

**Authors:** Xia Fang, Ximing Liao, Wei Gao, Wei Pei, Wujian Xu, Yuanyuan Wang, Yin Wu, Rongzhang Chen, Jiajun Ding, Muyun Wang, Xingyan Zhu, Heng Zhang, Dan-ni Ren, Qiang Li, Shaoyong Gao

**Affiliations:** 1 Department of Respiratory and Critical Care Medicine, Shanghai East Hospital, School of Medicine, Tongji University, Shanghai, China; 2 Department of Respiratory and Critical Care Medicine, Yingtan Hospital of Traditional Chinese Medicine, Yingtan, China; 3 Department of Respiratory and Critical Care Medicine, Yancheng Hospital of Traditional Chinese Medicine, Nanjing University of Chinese Medicine, Yancheng, China; 4 Academy of Integrative Medicine, College of Integrative Medicine, Fujian Key Laboratory of Integrative Medicine on Geriatric, Fujian University of Traditional Chinese Medicine, Fuzhou, China

**Keywords:** characteristics, complement system, neutrophil extracellular trap formation, proteomics, relapsing polychondritis, respiratory involvement

Authors “Xia Fang, Ximing Liao and Wei Gao” were erroneously omitted as equal contributing authors.

The original version of this article has been updated.

