# Supplementary material for: Unveiling the clinical and proteomic spectrum of relapsing polychondritis with respiratory involvement
Source: Front Immunol. 2026 Jul 17;17:1866652. doi: 10.3389/fimmu.2026.1866652 (PMC13469874; doi:10.3389/fimmu.2026.1866652)
Supplement: Supplementary file 1 [file Table1.docx]

Supplementary Table 1 The features of CT scan findings

|  | Number of patients | % of patients |
| --- | --- | --- |
| Subglottic stenosis | 31 | 40.26 |
| Tracheal Calcification | 50 | 64.94 |
| Tracheal Stenosis | 68 | 88.31 |
| Tracheal Thickening | 71 | 92.21 |
| Bronchial Calcification | 36 | 46.75 |
| Bronchial Stenosis | 76 | 98.70 |
| Bronchiectasis | 31 | 40.26 |
| Gas trapping | 31 | 40.26 |
| PET Scan | 14 | 18.18 |

Supplementary Table 2 The bronchoscopic findings

|  | Number of patients | % of patients |
| --- | --- | --- |
| Vocal cord oedema | 24 | 31.17 |
| Laryngeal cartilage deformation | 23 | 29.87 |
| Subglottic stenosis | 32 | 41.56 |
| mucosal hypertrophy | 69 | 89.61 |
| Disappearance of the cartilaginous rings | / | / |
| - trachea | 59 | 76.62 |
| - Bronchi above the lobar level | 75 | 97.40 |
| - Bronchi below the lobar level | 49 | 63.64 |
| Lumen softening and stenosis | / | / |
| - trachea | 64 | 83.12 |
| - Bronchi above the lobar level | 75 | 97.40 |
| - Bronchi below the lobar level | 49 | 63.64 |

Supplementary Table 3 Therapy

| Drug | Number of patients | % of patients |
| --- | --- | --- |
| Glucocorticoid |  |  |
| - Methylprednisolone | 30 | 38.96 |
| - Prednisone | 41 | 53.25 |
| DMARDs |  |  |
| - Methotrexate | 27 | 35.06 |
| - MMF | 11 | 14.29 |
| - Leflunomide | 2 | 2.60 |
| - Tacrolimus | 2 | 2.60 |
| - Azathioprine | 6 | 7.79 |
| - Cyclosporine | 3 | 3.90 |
| - Cyclophosphamide | 6 | 7.79 |
| Biologics |  |  |
| - Tofacitinib | 9 | 11.69 |
| - Baricitinib | 1 | 1.30 |
| - Abatacept | 1 | 1.30 |
| - Tocilizumab | 20 | 25.97 |
| - Adalimumab | 4 | 5.19 |

Supplementary Table 4 Control rate of biological agents

| Items | Tofacitinib | Tocilizumab | Adalimumab |
| --- | --- | --- | --- |
| Ear chondritis |  |  |  |
| - uncontrol,no.(%) | / | / | 1(100.00) |
| - control, no. (%) | 2(100.00) | 6(100.00) | / |
| Nasal chondritis |  |  |  |
| - uncontrol,no.(%) | / | / | / |
| - control, no. (%) | 3(100.00) | 7(100.00) | / |
| Laryngeal and Respiratory chondritis |  |  |  |
| - uncontrol,no.(%) | 6(75.00) | 14(66.67) | 4(100.00) |
| - control, no. (%) | 2(25.00) | 7(33.33) | / |
| Costochondritis |  |  |  |
| - uncontrol,no.(%) | 1(20.00) | 2(15.38) | / |
| - control, no. (%) | 4(50.00) | 11(84.62) | 1(100.00) |
| Ocular involvement |  |  |  |
| - uncontrol,no.(%) | / | / | 1(50.00) |
| - control, no. (%) | 1(100.00) | 3(100.00) | 1(50.00) |
| Joint involvement |  |  |  |
| - uncontrol,no.(%) |  |  |  |
| - control, no. (%) | 2(100.00) | 7(100.00) | / |
| Alive,no.(%) | 7(87.50) | 21(100.00) | 4(100.00) |
| Dead,no.(%) | 1(12.50) | 0(0.00) | 0(0.00) |

Supplementary Table 5 Clinical relevance of C3, C5 and MPO

| Characteristic | C3 | | P-value | C5 | | P-value |
| --- | --- | --- | --- | --- | --- | --- |
|  | Low (n=14) | High (n=20) |  | Low (n=18) | High (n=17) |  |
| Gender |  |  | 1.000 |  |  | 0.086 |
| - Male, no. (%) | 5  (35.71) | 7  (35.00) |  | 4 (22.22) | 9 (52.94) |  |
| - Female, no. (%) | 9  (64.29) | 13  (65.00) |  | 14 (77.78) | 8 (47.06) |  |
| Age at diagnosis |  |  | 0.364 |  |  | 0.691 |
| - ≤ 65 years, no. (%) | 13 (92.86) | 15 (75.00) |  | 15 (83.33） | 13 (76.47) |  |
| - > 65 years, no. (%) | 1 (7.14) | 5 (25.00) |  | 3 (16.67) | 4 (23.53) |  |
| Ear chondritis, no. (%) | 2 (14.29) | 3 (15.00) | 1.000 | 3 (16.67) | 4 (23.53) | 0.691 |
| Nasal chondritis, no. (%) | 4 (28.57) | 7 (35.00) | 1.000 | 5 (14.29) | 8 (47.06) | 0.305 |
| Laryngeal chondritis, no. (%) | 4 (28.57) | 9 (45.00) | 0.477 | 8 (27.78) | 7 (41.18) | 1.000 |
| Ocular involvement, no. (%) | 2 (14.29) | 4 (20.00) | 1.000 | 3 (16.67) | 4 (23.53) | 0.691 |
| Joint involvement, no. (%) | 2 (14.29) | 6 (30.00) | 0.422 | 4 (22.22) | 6 (35.29) | 0.471 |
| Cardiovascular, no. (%) | 0 (0.00) | 1 (5.00) | 1.000 | 0 (0.00) | 1 (5.88） | 0.486 |
| Respiraotry |  |  | 0.302 |  |  | 0.400 |
| - Central aiway ,no. (%) | 3 (8.82) | 6 (17.65) |  | 7 (20.00) | 3 (8.57) |  |
| - Central and peripheral aiway, no. (%) | 8 (23.53) | 6 (17.65) |  | 6 (17.14) | 8 (22.86) |  |
| - entire airway, no. (%) | 3 (8.82) | 8 (23.53) |  | 5 (14.29) | 6 (17.14) |  |
| Diagnostic delay, days | 163.00  (30.00, 337.50) | 220.50  (33.50, 545.00) | 0.389 | 195.00  (40.50, 496.25) | 150.00  (30.00, 390.00) | 0.507 |
| CRP, mg/L | 4.28  (2.63, 25.41) | 8.74  (3.77, 31.34) | 0.484 | 8.17  (3.40, 46.36) | 5.53  (2.45, 21.35) | 0.373 |
| ESR, mm/H | 23.50  (7.25, 36.25) | 27.50  (5.25, 52.50) | 0.752 | 21.00  (5.00, 54.50) | 25.00  (14.00, 33.00) | 0.921 |

(Continued from the previous table)

| Characteristic | MPO | | P-value |
| --- | --- | --- | --- |
|  | Low (n=17) | High (n=18) |  |
| Gender |  |  | 1.000 |
| - Male, no. (%) | 6 (35.29) | 7 (38.89) |  |
| - Female, no. (%) | 11 (64.71) | 11 (61.11) |  |
| Age at diagnosis |  |  | 0.228 |
| - ≤ 65 years, no. (%) | 12 (70.59) | 16 (88.89) |  |
| - > 65 years, no. (%) | 5 (29.41) | 2 (11.11) |  |
| Ear chondritis, no. (%) | 5 (29.41) | 2 (11.11) | 0.228 |
| Nasal chondritis, no. (%) | 7 (41.18) | 6 (33.33) | 0.733 |
| Laryngeal chondritis, no. (%) | 8 (47.06) | 8 (44.44) | 1.000 |
| Ocular involvement, no. (%) | 2 (11.76) | 5 (27.78) | 0.402 |
| Joint involvement, no. (%) | 7 (41.18) | 3 (16.67) | 0.146 |
| Cardiovascular, no. (%) | 0 (0.00) | 1 (5.56) | 1.000 |
| Respiraotry |  |  | 0.581 |
| - Central aiway, no. (%) | 6 (17.14) | 4 (11.43) |  |
| - Central and peripheral aiway, no. (%) | 5 (14.29) | 8 (22.86) |  |
| - entire airway, no. (%) | 6 (17.14) | 6 (17.14) |  |
| Diagnostic delay, days | 175.00  (30.00, 390.00) | 195.00  (30.00, 592.50) | 0.691 |
| CRP, mg/L | 9.67  (3.18, 29.07) | 6.35  (3.40, 24.85) | 0.766 |
| ESR, mm/H | 25.00  (8.50, 42.50) | 20.00  (7.25, 53.25) | 0.843 |
